# Evaluation of oxfendazole in the treatment of zoonotic *Onchocerca lupi* infection in dogs

**DOI:** 10.1371/journal.pntd.0006218

**Published:** 2018-01-29

**Authors:** Vito Colella, Carla Maia, André Pereira, Nuno Gonçalves, Marta Caruso, Coralie Martin, Luís Cardoso, Lenea Campino, Ivan Scandale, Domenico Otranto

**Affiliations:** 1 Dipartimento di Medicina Veterinaria, Università degli Studi di Bari, Str. Prov. per Casamassima km 3, Valenzano, Italy; 2 Global Health and Tropical Medicine (GHTM), Instituto de Higiene e Medicina Tropical (IHMT), Universidade Nova de Lisboa (UNL), Rua de Junqueira 100, Lisboa, Portugal; 3 Hospital Veterinario do Algarve, Servico de Ecografia veterinaria ambulatoria, Avenida Calouste Gulbenkian, Faro, Portugal; 4 Istituto Zooprofilattico Sperimentale della Puglia e della Basilicata, Via della Tecnica 23, Matera, Italy; 5 Unité Molécules de Communication et Adaptation des Microorganismes, Sorbonne Universités, Muséum National d'Histoire Naturelle, Rue Cuvier, Paris, France; 6 Department of Veterinary Sciences, University of Trás-os-Montes e Alto Douro (UTAD), Vila Real, Portugal; 7 Drugs for Neglected Diseases *initiative*, Chemin Louis-Dunant, Geneva, Switzerland; McGill University, CANADA

## Abstract

The genus *Onchocerca* encompasses parasitic nematodes including *Onchocerca volvulus*, causative agent of river blindness in humans, and the zoonotic *Onchocerca lupi* infecting dogs and cats. In dogs, *O*. *lupi* adult worms cause ocular lesions of various degrees while humans may bear the brunt of zoonotic onchocercosis with patients requiring neurosurgical intervention because of central nervous system localization of nematodes. Though the zoonotic potential of *O*. *lupi* has been well recognized from human cases in Europe, the United States and the Middle East, a proper therapy for curing this parasitic infection in dogs is lacking. To evaluate the efficacy of oxfendazole, 11 out of the 21 client-owned dogs (21/123; 17.1%) positive for skin-dwelling *O*. *lupi* microfilariae (mfs), were enrolled in the efficacy study and were treated with oxfendazole (50 mg/kg) per OS once a day for 5 (G2) or 10 (G3) consecutive days or were left untreated (G1). The efficacy of oxfendazole in the reduction of *O*. *lupi* mfs was evaluated by microfilarial count and by assessing the percentage of mfs reduction and mean microfilaricidal efficacy, whereas the efficacy in the reduction of ocular lesions was evaluated by ultrasound imaging. All dogs where subjected to follow-ups at 30 (D30), 90 (D90) and 180 (D180) days post-treatment. The percentage of reduction of mfs was 78% for G2 and 12.5% for G3 at D180. The mean microfilaricidal efficacy of oxfendazole in the treatment of canine onchocercosis by *O*. *lupi* at D30, D90 and D180 was 41%, 81% and 90%, in G2 and 40%, 65% and 70%, in G3, respectively. Retrobulbar lesions did not reduce from D0 to D180 in control group (dogs in G1), whereas all treated dogs (in G2 and G3) had slightly decreased ocular lesions. Percentage of reduction of ocular lesions by ultrasound examination was 50% and 47.5% in G2 and G3 at D180, respectively. Despite the decrease in ocular lesions in all treated dogs (G2 and G3), oxfendazole was ineffective in reducing ocular lesions and skin-dwelling *O*. *lupi* mfs in treated dogs (G2 and G3) in a six-month follow-up period. Here we discuss the need for more reliable diagnostic techniques and efficient treatment protocols to better plan future intervention strategies.

## Author summary

The genus *Onchocerca* (Spirurida, Onchocercidae) includes *Onchocerca volvulus*, which is estimated to infect at least 37 million people globally, and zoonotic *Onchocerca lupi* in carnivores. Infection by *O*. *lupi* has been reported in dogs and cats from several European countries and recently also in the U.S. and Canada, causing mainly ocular lesions. In humans, *O*. *lupi* displays a marked neurotropism with nematodes embedded in nodules localized in the cervical spine of infant, children and adults. Though the reported severity of infection in humans and the high prevalence detected in dogs are now well-recognized, a proper treatment regime for curing this parasitic infection is lacking, being the surgical removal of the parasitic nodule the therapy of choice in canine patients. Hence, there is an unmet medical need for treatment of this zoonotic disease in both humans and animals. In this study we evaluated the efficacy of oxfendazole under two treatment regimes in the reduction of ocular lesions and skin-dwelling microfilariae of *O*. *lupi* in naturally infected dogs.

## Introduction

The genus *Onchocerca* (Spirurida, Onchocercidae) encompasses parasitic nematodes mainly associated to ungulates, with the exception of *Onchocerca volvulus* in humans and *Onchocerca lupi* in carnivores [1–3]. While *O*. *volvulus* is a well-known parasite estimated to infect 37 million people globally (www.cdc.gov/globalhealth/ntd/diseases/oncho_burden.html), infection by *O*. *lupi* has been reported from dogs and cats in Hungary, Greece, Germany, Portugal, Romania and Spain [4–11], and also in the U.S. and Canada [12–18]. Adult *O*. *lupi* are found within nodules embedded in ocular tissues and annexes [5, 15, 17], and such presentation commonly leads to clinical diagnosis. However, infections may be associated with no clinical signs [19], to severe ocular disease, including blindness [20]. Nonetheless, the limited number of case reports hinders a thorough understanding of the pathogenesis of canine onchocercosis, including cases with *O*. *lupi* in the retrobulbar space of the eye, with no overt sign of infection [15]. In the latter case, imaging techniques (i.e. ultrasound scans and computed tomography [21]), or the detection of microfilariae (mfs) in skin snips [14] are the only diagnostic tools available. For instance, an overall prevalence of infection by *O*. *lupi* of 8.4% was recorded by mfs counts in skin biopsy sediments in apparently healthy dogs from Greece and Portugal [19].

Humans may bear the brunt of zoonotic onchocercosis by *O*. *lupi* with patients requiring neurosurgical intervention because of nematode localization in the cervical spine of an infant [22] and children [16, 23], thus making central the development of treatment strategies of reservoir animals. However, though the zoonotic potential of *O*. *lupi* has been well recognized from cases reported in Europe, the United States and Middle East [15, 16, 24], scientific knowledge on the biology, pathogenesis and treatment of this parasitosis is minimal. A proper treatment regimen for curing this parasitic infection is lacking and the surgical removal of the parasitic nodule has been the therapy of choice in canine patients.

Drug-based treatments included various combination and dosages of melarsomine, ivermectin, topical and systemic antibiotics and prednisone [15, 17, 25]. However, proper studies on the long-term outcomes of these therapies have not yet been performed, and there is an urgent need for studies assessing the efficacy of molecules during natural infection with *O*. *lupi* in dogs.

Benzimidazole (BDZ) drugs have a broad-spectrum activity and low toxicity, and have been approved, more than 30 years ago, in human and veterinary medicine against several helminth species, including gastrointestinal parasites and lungworm infections in animals. In this class of drugs, oxibendazole and oxfendazole (OXF) have been increasingly tested as anthelmintics used in human medicine, for their potential efficacy also against tissue-dwelling larval helminths [26, 27].

In addition, benzimidazole drugs (flubendazole, mebendazole, OXF, albendazole, fenbendazole) have shown an *in vivo* macrofilaricidal activity against several filarial species in animal models [27]. In particular, their efficacy was assessed against larval and adult forms of *Brugia malayi*, *Brugia pahangi*, *Acanthocheilonema viteae* and, *Litomosoides sigmodontis*, in experimentally infected rodents [28–32]. Significant effects on the microfilaremia after treatment are not always correlated with adulticidal efficacy suggesting that subdoses may alter embryogenesis. However, differences in efficacy of benzimidazoles have been related to the parasite species and the route of drug administration. By subcutaneous administration, OXF has shown either no activity [33] or full protection against adults of *B*. *pahangi* [34] whereas it exhibited a marked macrofilaricidal activity against *L*. *sigmodontis* [33].

In this study we evaluated the efficacy of OXF under two treatment regimens in the reduction of ocular lesions and skin-dwelling mfs of *O*. *lupi* in naturally infected dogs.

## Methods

### Ethics statement

This study was performed as a negative controlled, blinded and randomised field study in privately owned dogs conducted according to the principles of Good Clinical Practices (VICH GL9 GCP) [35]. The protocol of this study was approved by the Ethical Committee of the Department of Veterinary Medicine of the University of Bari (Prot. Uniba 1/16). All dogs were living in an *O*. *lupi* endemic area of Algarve region (southern Portugal [19]), and the study procedures on animals were performed after receiving the owner’s informed consent.

In October 2016, privately owned dogs (n = 123) were sampled via skin snip and positive animals were subjected to ultrasound examination for diagnosing *O*. *lupi* infection. All animals lived in the municipalities of Tavira, Faro and Castro Marim.

### Skin sampling

Skin samples were collected in the afternoon-evening by using a disposable punch over an area of ≈0.4 × 0.5 cm from the interscapular regions of the dogs [19]. Skin biopsies (one per dog at each time point) were soaked in saline solution for 12 h at room temperature and sediments (20 μL) were individually observed under light microscopy. Microfilariae were identified according to morphological keys [19], and their numbers were assessed from each positive animal by a blinded double-check counting of two independent operators.

### Molecular identification

Microfilariae were isolated and genomic DNA extracted using a commercial kit (DNeasy Blood & Tissue Kit, Qiagen, Germany) in accordance with the manufacturer’s instructions. Samples were molecularly processed for specific amplification and sequencing of the partial cytochrome oxidase subunit 1 (*cox*1) gene (~689 bp), following procedures described elsewhere [36]. Amplicons obtained from the skin sediments were purified using Ultrafree-DA columns (Amicon, Millipore, USA) and sequenced directly with the Taq DyeDeoxyTerminator Cycle Sequencing Kit (v.2, Applied Biosystems, USA) in an automated sequencer (ABI-PRISM 377, Applied Biosystems).

Sequences were aligned using the Geneious R9 software package (http://www.geneious.com) and compared (BLASTn) with those available in the GeneBank database (http://blast.ncbi.nlm.nih.gov/Blast.cgi).

### Ultrasound examination

Following the assessment of mfs counts, each positive animal was checked by an ultrasound examination of both eyes and retrobulbar spaces as follow. Briefly, 1–2 minutes prior to the exam oxibuprocain chloridrate (Anestocil, Laboratório Edol, Portugal) was used as local anaesthetic, placing a few drops on each eye, with animals restrained in sternal recumbence. The eye and the retrobulbar space were scanned with a portable ultrasound Logic Book–GE, equipped with two probes, one linear and one microconvex, with frequency ranges from 6–10 MHz, through transcorneal, transscleral and transpalpebral approaches, along horizontal, vertical and oblique planes to check for lesions associated with the presence of adult parasites.

### Enrolment and follow up

Naturally infected dogs were enrolled in the study if scored positive for *O*. *lupi* mfs at skin sediment observation and further molecular diagnostic confirmation. Nodules or hyper-echogenic lesions caused by adult nematodes were also assessed. Dogs were allocated to study groups in blocks following a random treatment allocation plan on the basis of an inclusion sequence. Each dog, per block, was randomly assigned to one untreated control group (G1) and to treated groups (G2 and G3). Animals were treated with Dolthene (Boheringer Ingelheim, Germany) a commercial oral suspension of OXF for dogs containing 22.65 mg/ml of OXF, 1.5mg/ml of sorbic acid (E200), macrogol, macrogol stearate, sodium carboxymethyl cellulose, silica colloidal anhydrous, citric acid monohydrate (E330), sodium citrate and purified water. A dose of 50 mg/kg per body weight per OS once daily for 5 (G2) or 10 (G3) consecutive days was administrated. No information is available on the in vitro activity of OXF against *O*. *lupi* microfilaria or adult parasites. Furthermore in vitro activity of anthelmintic benzimidazoles in general is difficult to be assessed [37, 38]. Therefore, a relevant drug concentration to be achieved in plasma of infected animals cannot derive from in vitro efficacy studies. To maximise the chance of observing a pharmacological effect a high dose of 50mg/kg/day for 5 (G2) and 10 (G3) consecutive days was selected to achieve a plasma disposition of OXF during the whole treatment duration above approximately 1 μg/ml [39]. Efficacy of the treatment was assessed by microfilarial count and presence and size of ocular nodules, at 30 (D30), 90 (D90) and 180 (D180) days post-treatment.

### Assessment of the efficacy and statistical analyses

The percentage (%) of reduction [s] of ocular lesions was calculated as follow [s] = [(Cs0 –Cs) / Cs0] x 100, where Cs0 is the baseline ocular size lesion before treatment and Cs was the count at Cs was the count at any time point (s). The percentage (%) of reduction [t] of mfs was calculated as follow [t] = [(Ct0 –Ct) / Ct0] x 100, where Ct0 is the baseline count before treatment and Ct was the count at any time point (t). Moreover, mean microfilaricidal efficacy (%) = [(Ct–T) / Ct] x 100, where Ct is the mean count of mfs of the control group at X time and T is the mean count of mfs of the treated animal groups at X time. The significance of the mfs count reduction and mean microfilaricidal efficacy in treated dogs was analysed by ANOVA, with standard statistical assumption. Statistical analysis was planned and conducted in compliance with current guidelines [40]. Statistical calculations and randomization were performed with SPSS statistical package for Windows, version 13.0, and nQuery+nTerim 3.0 (StatSols), Statistical Solutions Ltd. 2014, Microsoft. A post-hoc power calculation on the mfs counts and ocular lesions at day 0 and at the several days of measurement has been calculated by the software GPower 3.1.9.2, using the module F-test, ANOVA model for repeated measures with between-within factor interaction, setting the power at 80% and significance level at 0.05 and the sample size was evaluated in function of effect size.

### Pharmacokinetic of oxfendazole

In order to assess the pharmacokinetic of OXF, and the metabolites fenbendazole (FBZ) and fenbendazole-sulfone (FBZSO2), heparinised blood samples were collected by cephalic vein puncture prior to the start of treatment from all dogs (G1, G2 and G3 groups) and, once a day, at different time points (i.e., +1, +5, +6, +7 day post treatment (pt) for G2, and +1, +5, +10, +11 and +12 day pt for G3). Samples were collected immediately prior to the daily drug administration.

A 20 μL samples of whole blood sample from each animal was transferred into 96-well polypropylene plate and added with 20 μL of blank dog plasma. After mixing a volume of 400 μL of acetonitrile was added to each well. The plate was than mixed and centrifuged at 2100 *g* for 20 min and 2 μL of supernatant was directly injected in the LC-MS/MS system. Calibration standards in the range of 1 to 5000 ng/mL and added with 20 μL of blank dog whole blood were included in duplicate at each run.

OXF, FBZ and FBZSO2 were detected and quantified using an Agilent 1100 series HPLC connected to a API4000 QTRAP Mass Spectrophotometer (SCIEX, Applied Biosystems, USA). Chromatographic separation was achieved using a Kinetex C18 analytical column (50*3.0mm, 2.6 mm. Phenomenex, USA) column maintained at 40°C and eluted with a gradient of ammonium acetate (10 mM) and acetonitrile. The run time was 0.6 min. The detection and quantification of the three compounds was performed in the tandem mass spectrometry operated in positive electro-spray ionisation and multiple reaction monitoring mode using the transition range of *m/z* 316–191.2 for OXF, 300.1–268.2 for FBZ and 332.1–300.3 for FBZSO2. The ion source and gas parameters were: curtain gas 20 psi, ion source gas 45 (GS1) and 40 (GS2), source temperature 450°C and collision gas set to medium. The optimized acquisition parameters for the three analytes were: declustering potential 95 V for OXF and FBZSO2 and 120 V for FBZ); entrance potential 10 V for all analytes; collision energy 30 V, 28 V and 31 V for OXF, FBZ and FBZSO2, respectively and collision cell exit potential 15 V for OXF and f FBZSO2 and 10 V for FBZ.

The performance of the LC-MS/ MS method was tested using a short validation protocol. Linearity of calibration was confirmed at concentrations ranging from 2.5 to 5000 ng/mL (r^2^ = 0.9975) for OXF, from 1.0 to 1000 ng/mL (r^2^ = 0.9964) for FBZ and from 1.0 to 2500 ng/mL (r^2^ = 0.995) for FBZSO2. The mean extraction recovery was not less than 80% for all analytes tested and the accuracy (expressed as %Bias) and precision (expressed as % CV) of the method ranged from -2.9 to 3.4% and 2.5 to 6.7% respectively for OXF, from -1.6 to 2.0% and 2.8 to 6.4% for FBZ and from -10.4 to 6.4%, and 0.1 to 9.0% for FBZSO2. The lower limit of quantification (LLOQ) was 2.5 ng/mL for OXF and 1.0 ng/mL for FBZ and FBZSO2. Results were expressed as mean (± s.e.m.). Differences in drug blood concentration at different sampling times were determined using one-way ANOVA and results were considered statistically significant when *p*<0.05.

## Results

Of the 123 animal sampled, 21 (17.1%) scored positive for *O*. *lupi* mfs, out of which 11 were enrolled in the efficacy study and assigned to groups G1 (dogs 1–4), G2 (dogs 5–8) and G3 (dogs 9–11). The mean number of *O*. *lupi* microfilariae in skin sediment was homogenous amongst the three groups (*p*<0.05). The morphological identification of mfs was molecularly confirmed, and nucleotide sequences obtained from mfs DNA (GenBank; accession number: MG677940) displayed 100% identity with those of *O*. *lupi* from Portugal (GenBank; accession number: EF521410).

### Efficacy study

The count number of *O*. *lupi* mfs at each study day is reported in Table 1. The percentage of reduction of mfs was 78% for G2 and 12.5% for G3 at D180. Mean microfilaricidal efficacy of OXF in the treatment of canine onchocercosis by *O*. *lupi* was 41%, 81% and 90%, respectively at D30, D90 and D180 in G2 compared to G1 and 40%, 65% and 70%, respectively at D30, D90 and D180 in G3 compared to G1. Differences in mean microfilaricidal efficacy in animals in G2 and G3 compared to the control group (G1) were not statistically significant at all time points, except at D90 between G2 and control group (*p*<0.05).

**Table 1:** Number of skin-dwelling microfilariae detected from dogs in the control and treatment groups.

Study group	Serial number of dogs	D0	D30	D90	D180
G1	1	23	4	7	5
	2	10	7	11	168
	3	2	6	6	5
	4	19	34	18	43
G2	5	95	26	5	8
	6	1	0	0	0
	7	2	4	3	14
	8	1	0	0	0
G3	9	35	18	9	29
	10	20	1	0	18
	11	1	4	2	2

Number of microfilariae detected from 20 μL of skin sediment of dogs in the control (G1) and treatment (G2 and G3) groups at 0 (D0), 30 (D30), 90 (D90) and 180 (D180) days from the enrolment.

**Fig 1:**
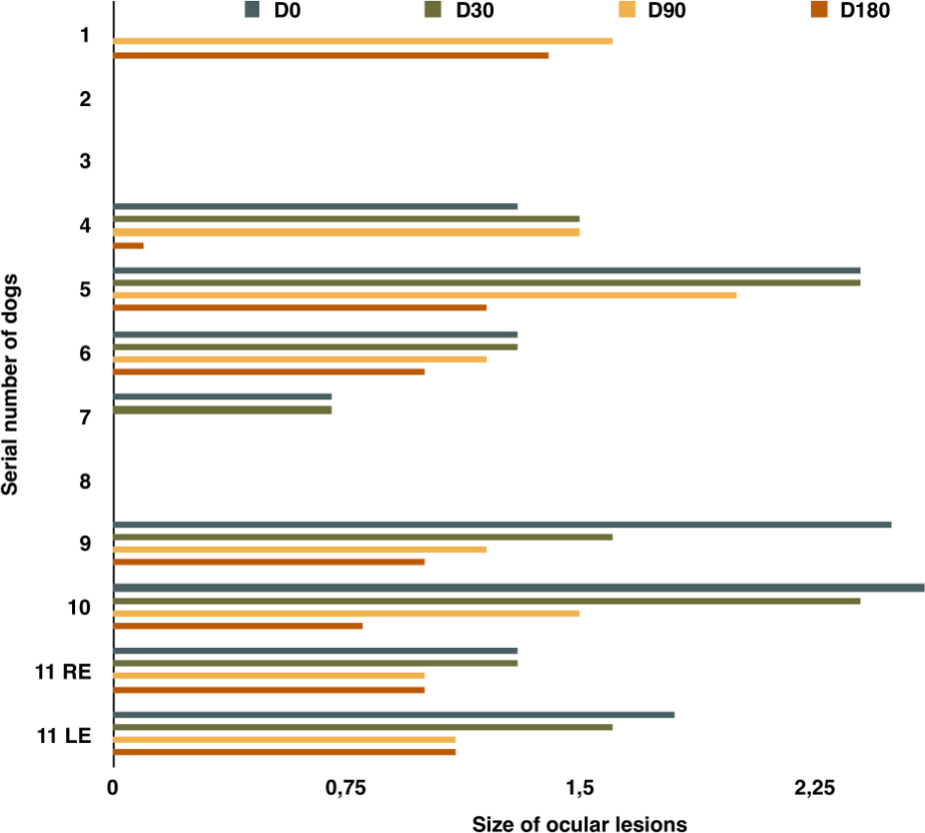
Size of ocular lesions in dogs enrolled in the efficacy study. Size of ocular lesions detected at the ultrasound imaging of eyes of dogs at 0 (D0), 30 (D30), 90 (D90) and 180 (D180) days post enrolment in animals in G1 (1–4), G2 (5–8) and G3 (8–11). RE = Right Eye; LE = Left Eye.

**Fig 2:**
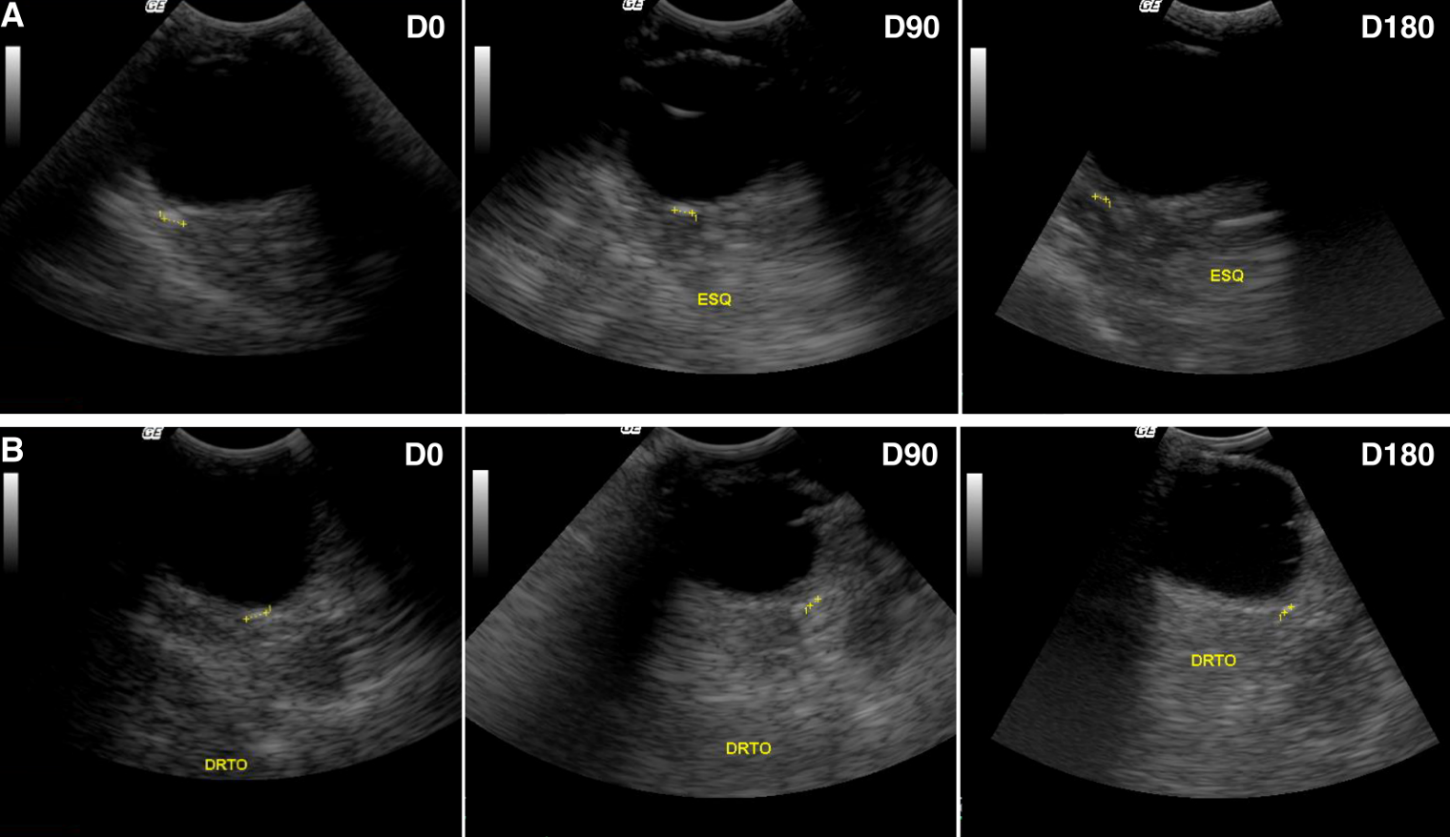
Decreased ocular size lesions in dogs treated with oxfendazole. Decreased ocular size lesions in dogs n. 5 (A) and n. 9 (B) at 0 (D0), 90 (D90) and 180 (D180) days post enrolment.

On D0, eight dogs (i.e. nos. 1, 4, 5, 6, 7, 9, 10, 11) had hyper-echogenic lesions (0.7–2.5 mm wide) in the retrobulbar space overlapping the localization and the dimensions of *O*. *lupi* adult nematodes. At the ultrasound examination, retrobulbar lesions did not reduce from D0 to D180 in dogs of the G1, whereas one dog of G2 (no. 7) cleared the ocular lesion and all the other dogs of treatment groups had a slightly decreased size of ocular lesions (Figs 1 and 2). Percentage of reduction of ocular lesions by ultrasound examination was not statistically significant, being of 50% and 47.5% at D180 in G2 and G3, respectively.

### Pharmacokinetic results

**Fig 3:**
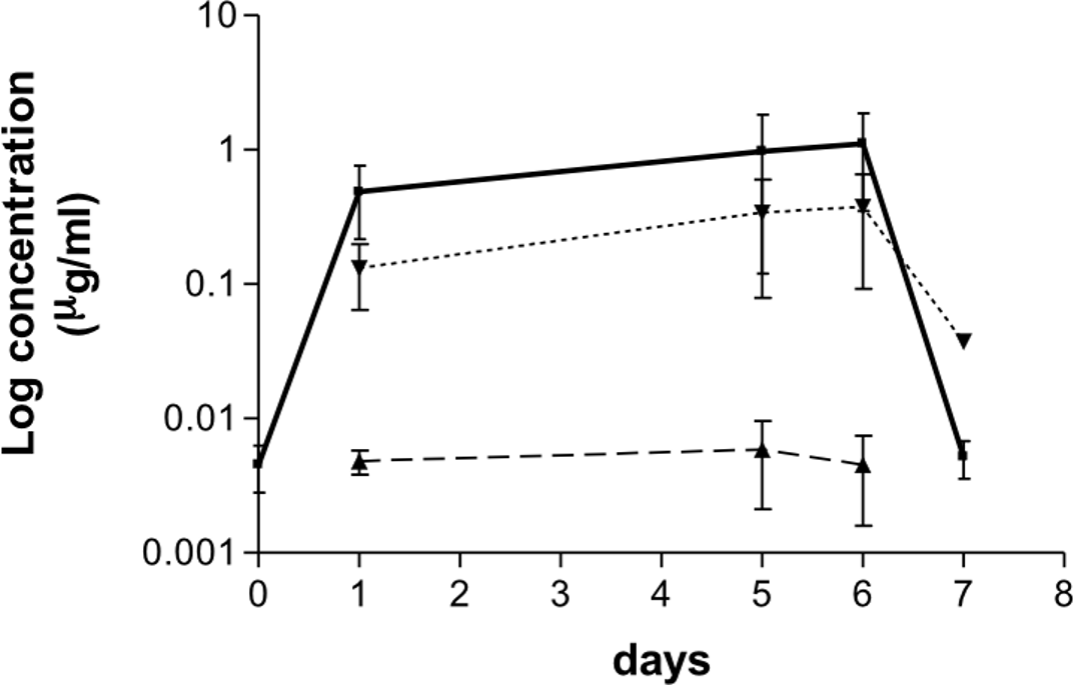
Pharmacokinetic of oxfendazole in dogs treated for 5 days with oxfendazole. Mean (± s.e.m.) blood concentrations (μg/mL) of oxfendazole (square) and its metabolites fenbendazole (triangle) and fenbendazole-sulfone (triangle) following oral administration of 50 mg/kg b.w. for 5 days in dogs (n = 4).

**Fig 4:**
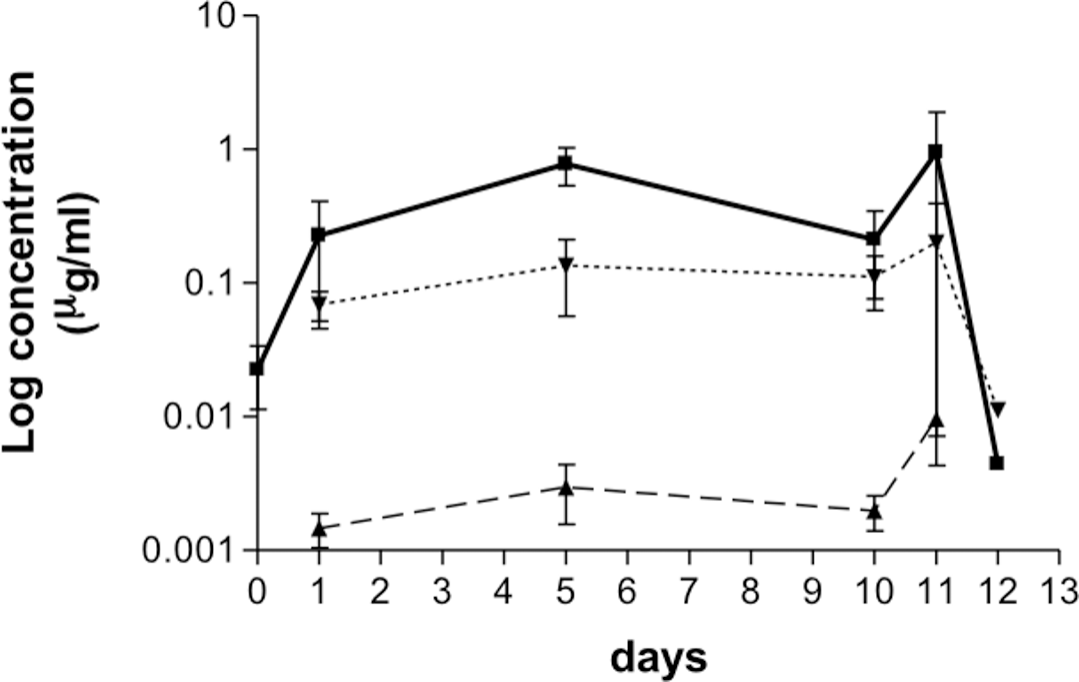
Pharmacokinetic of oxfendazole in dogs treated for 10 days with oxfendazole. Mean (± s.e.m.) blood concentrations (μg/mL) of oxfendazole (square) and its metabolites fenbendazole (triangle) and fenbendazole-sulfone (triangle) following oral administration of 50 mg/kg b.w. for 10 days in dogs (n = 3).

All samples collected from untreated dogs were below the limit of detection of the method. The plasma concentrations time curves of OXF and its metabolites following oral administration of 50 mg/kg for 5 or 10 days are shown in Figs 3 and 4. At the zero-time point, prior to the first administration, the mean of OXF plasma levels is above zero, this likely caused by a carryover effect. Standard deviations of measured plasma levels indicate high variability from day 1 to day 5 and day 10, while OXF is administrated to animals. Among others, the heterogeneity of treated animals, in regards of species, weight and age is likely to be the cause for such variability. Over the 5-days period blood mean concentration of OXF varied at trough level 0.49±0.24 μg/mL on day 1 to 0.98±0.74 μg/mL on day 5 and over the 10-days period from 0.23±0.13 μg/mL on day 1 to 0.78±0.17 μg/mL on day 5. By 1 day after the last dosing (day 6 and day 11, respectively) the drug blood concentrations were maintained at similar levels than those recorded during the treatment and they fell to 0.005±0.002 μg/mL and 0.003±0.001 μg/mL 2 days after the last drug administration (day 7 and day 12 respectively). No significant differences among the blood drug concentrations were recorded from the first day treatment to the first day post-treatment (one-way ANOVA). In conclusion, the overall exposure of OXF was sustained in all animals during the whole treatment duration. In both administration protocols, the blood concentration of FBZSO2 followed a similar but lower pattern to OXF. Over the 5-days period, the mean concentration percentage of FBZSO2 compare to OXF varied from 27% on day 1 to 34% on day 6; over the 10-days period, a similar variation is observed: 31% on day 1, to 17% on day 5 and to 31% on day 11. The metabolic biotrasformation of the parent drugs into the reduced metabolites fenbendazole was negligible.

## Discussion

In this study, OXF was ineffective in reducing ocular lesions and skin-dwelling mfs of *O*. *lupi* in naturally infected dogs in a six-month follow-up period. Though one treated dog cleared the ocular lesions, OXF did not show any major efficacy in reducing the size of hyper-echogenic areas in the retrobulbar space. Ultrasound examination has several limitations in the detection of the parasites, including lack of sensitivity and specificity, but represents the only option available to detect nematodes with retrobulbar localization [21].

Only at D90, but not D180, OXF showed a significant efficacy in reducing the number of mfs in animals from G2 compared to the control group. Moreover, the increased number of mfs in 6 out of 11 dogs from D30 to D180 together with the lack of significant decrease of size of ocular lesions stands for a lack of efficacy in treatment of canine onchocercosis. In addition, this variation in number of mfs from November (D30) to April (D180) may indicate the existence of a seasonal pattern, which may match with the behaviour of the as-yet-unknown vector species. Up to now, the detection of DNA in wild-caught *Simulium tribulatum* indicates this simulid species as putative vector in California [41], though further studies are needed to ascertain species of vectors involved in the epidemiology of *O*. *lupi*. The seasonal variation in *O*. *lupi* mfs would add further information to their circadian rhythm [42], also considering that seasonal pattern has been well studied in the life-cycles of *O*. *volvulus* and *Dirofilaria immitis* [43–46].

The highest percentage of reduction of mfs in dogs treated for 5 days rather than for 10 days (78% *vs* 12.5%) is unexpected. OXF is a macrofilaricide and the evaluation of its efficacy solely based on mfs counting may be troublesome, also considering that their lifespan in the definitive hosts is unknown. Moreover, the enrolment of naturally infected animals in this trial was challenging and resulted in a limited number of dogs included in the study. Indeed, it would have been desirable a large effect size (large difference between first observations and subsequent follow-ups) like the one observed, albeit variances of the model resulted very small to achieve 80% power, i.e. a higher level of probability that there is an effect if the differences are statistically significant, a greater sample size should be recruited, with a lower effect size that is still clinically interesting.

Nevertheless, benzimidazoles, including OXF, have been described, as tubulin inhibitors, preventing polymerisation of the tubulin subunit α and ß [47]. Though this mechanism of action is compatible with inhibition of the embryogenesis [48, 49], this effect did not seem to occur in the present study. Further explanations for the lack of efficacy of OXF may be the pharmacokinetic differences, though not statistically significant, in dogs from G2 and G3 (mean blood concentration 0.49–0.97 in G2 *vs* 0.23–0.78 μg/mL in G3). The concentration of OXF, FBZSO2 and FBZ, measured at the trough level is in the same range of previous assessment of the plasma disposition in dogs at the same dosage (i.e. 50mg/kg) after a single administration [39]. The metabolic biotransformation of OXF occurs via oxidation of the sulfoxide group of OXF to form FBZSO2. This is consistent with oral pharmacokinetic studies conducted in cattle, sheep, dog and pig, where such pattern was observed as well over a period of 24 hours [50]. However, this is not case in horses and rats where plasma concentration of OXF is less abundant compared to FBZ and FBZSO2 respectively, over a period of 24 hours [50]. In sheep, an equilibrium between FBZ and OXF has been described in vivo [51]. This was not observed in the current study (Figs 3 and 4) where formation of FBZ is negligible as reported by [39]. Following 5 and 10 consecutive days of drug administration, OXF blood concentration did not vary being almost undetectable after the second day from the last administration (i.e. at day 7 and day 12 in G2 and G3, respectively). This data may be beneficial for future efficacy studies and confirm the shorter blood retention time of OXF in dogs’ plasma (see also [39]).

Despite a sustained exposure of OXF was observed in all dogs in treatment group, the drug concentration in the nodules is unknown. Also, vascularization of *O*. *lupi* nodules is not described and it is possible that OXF plasma concentration is not representative of the drug concentration that will eventually reach the adult parasites. Although OXF is a broad spectrum anthelmintic effective for several filarial species [52], it may be not effective against *O*. *lupi*.

The percentage of dogs positive (17.1%) for *O*. *lupi* mfs represents the highest prevalence of canine onchocercosis detected, as in an epidemiological study performed in dogs from Algarve the 8.3% of the examined animals scored positive [19]. Remarkably, animals in the abovementioned survey and those in the present study were clinically healthy, drawing the attention on the role of dogs as suitable reservoir of *O*. *lupi* in endemic area.

The absence of palpable nodules together with the lack of ocular lesions at ultrasound examination in some of the study animals, which harboured mfs in their skins, imply the potential presence of adult nematodes in other anatomical localization. For instance, gravid females of *O*. *lupi* were found in the thyroid cartilage of a dog in Portugal, with no involvement of ocular tissues [53]. Additionally, in a retrospective study of cases from dogs in New Mexico (U.S.), the 67% of animals treated with melarsomine, ivermectin and doxycycline had recurrent ocular disease [17], which was in contrast with a similar study in Greece, where no recurrence was noticed after drug administration [5]. This apparent challenge in treating animals from the U.S. has been attributed to the presence of a single haplotype circulating in this country [17]. Again, humans infected by *O*. *lupi* from the U.S. displayed more severe diseases (e.g. spinal and orbital localization) but not subconjunctival nodules [16]. A recent phylogenetic analysis indicates that *Onchocerca* species form a monophyletic group encompassing three clades, one of which composed of *Onchocerca gutturosa*, *O*. *linealis* and *Onchocerca ochengi* of domestic bovids, *O*. *volvulus* of humans and *O*. *lupi* [3]. In addition *O*. *ochengi* and *O*. *volvulus* are sister species, with *O*. *lupi* being basal to this clade [3]. In that study based on a single-gene analysis *O*. *lupi* showed a large genetic intraspecific variability, suggesting the existence of two clades, one detected only from Portugal and all the others distributed in Europe and in the U.S. and this is consistent with either two- or seven-gene analysis [3, 13]. Nonetheless, epidemiological and clinical studies coupled with the genetic traits of *O*. *lupi* should be conducted to elucidate whether haplotypes occurring in different geographical areas could play a role in the disease ecology and treatment efficacy.

Non-surgical treatment strategies may include the use of microfilaricide and anti-symbiont drugs. For instance, among available pharmaceutical options ivermectin showed to be effective against *O*. *volvulus* mfs [54, 55].

With the exception of *Onchocerca flexuosa*, *Wolbachia* symbionts have been detected in all the species of the *Onchocerca* genus [56], including *O*. *lupi* [12, 57] making this species a potential focus for symbiont-targeted therapy. Among others, these bacteria favour the survival of the mfs and interact with the cell of the immune system modulating responses to inflammation. Hence, treatments aiming at *Wolbachia* results in sterilization and death of the adult worms and first-to-third larval moulting blockage [58]. Nonetheless, though the clade in which *O*. *lupi* clusters have the strongest co-evolutionary pattern with their *Wolbachia* symbionts [3], few studies included anti-*Wolbachia* treatments as potential target for therapy [17].

Undoubtedly, a defined treatment protocol for this infection is still lacking and the therapies employed up to now mostly derive from clinical experience on the treatment of heartworm disease in dogs [59, 60]. Infections with *O*. *lupi* can inflict important hardship on the health of people, and there is an unmet medical need for treatment of this zoonotic disease in both humans and animals. Future efficacy studies are, therefore, urgently needed and should take into account the difficulties in the detection of adult and larval stages of this zoonotic filarioid.
